# SOCS2 Is Critical for the Balancing of Immune Response and Oxidate Stress Protecting Against Acetaminophen-Induced Acute Liver Injury

**DOI:** 10.3389/fimmu.2018.03134

**Published:** 2019-01-22

**Authors:** Renata Monti-Rocha, Allysson Cramer, Paulo Gaio Leite, Maísa Mota Antunes, Rafaela Vaz Sousa Pereira, Andréia Barroso, Celso M. Queiroz-Junior, Bruna Araújo David, Mauro Martins Teixeira, Gustavo Batista Menezes, Fabiana Simão Machado

**Affiliations:** ^1^Department of Biochemistry and Immunology, Institute of Biological Sciences (ICB), Universidade Federal de Minas Gerais (UFMG), Belo Horizonte, Brazil; ^2^Department of Morphology, Institute of Biological Sciences (ICB), Universidade Federal de Minas Gerais (UFMG), Belo Horizonte, Brazil

**Keywords:** SOCS2, liver necrosis, immune response, oxidative stress, acetaminophen

## Abstract

Acetaminophen (APAP) is usually safe when administrated in therapeutic doses; however, APAP overdose can lead to severe liver injury. APAP can cause direct hepatocyte damage, and stimulates an inflammatory response leading to oxidative stress. Supressor of Cytokine Signaling (SOCS) 2 modulates cytokine and growth factor signaling, and plays a role in the regulation of hepatic cellular processes. Our study evaluated the role of SOCS2 in APAP liver injury. The administration of a toxic dose (600 mg/kg) of APAP caused significant liver necrosis in WT mice. In SOCS2^−/−^ mice, there was significantly more necrosis, neutrophil recruitment, and expression of the neutrophil-active chemokine CXCL-1. Expression of proinflammatory cytokines, such as TNF-α and IL-6, was elevated, while expression of anti-inflammatory cytokines, IL-10 and TGF-β, was diminished. *In vitro*, SOCS2^−/−^ hepatocytes expressed more p-NF-kB and produced more ROS than WT hepatocytes when exposed to APAP. SOCS2^−/−^ hepatocytes were more sensitive to cell death in the presence of IL-6 and hydrogen peroxide. The administration of catalase *in vitro* and *in vivo* resulted in a pronounced reduction of cells/mice death and necrosis in the SOCS2^−/−^ group. We have demonstrated that SOCS2 has a protective role in the liver by controlling pro-oxidative and inflammatory mechanisms induced by APAP.

## Introduction

Acetaminophen (APAP) has been used to treat pain and fever for more than 50 years. While it is safe and effective when used as directed, due to its relatively narrow therapeutic index and widespread availability, exceeding the recommended dose can lead to liver toxicity. For adults, a single dose of 10–15 g can cause hepatic necrosis, and for some individuals the toxicity threshold may be lower (1, 2). Currently, overdose of APAP is the most prevalent cause of acute liver failure in many countries, including the USA and the UK (3).

In hepatocytes, APAP is metabolized into N-acetyl-p-benzoquinone imine (NAPQI) mainly by cytochrome P450 2E1. Accumulated NAPQI depletes glutathione (GSH) and binds to mitochondrial proteins (4), which leads to inhibition of mitochondrial respiration (5, 6) resulting in reactive oxygen species (ROS) formation (7). In addition to NAPQI/ROS-induced hepatotoxicity, liver injury is propagated by an inflammatory response after APAP is metabolized (8). This triggers the innate immune response, including activation of neutrophils (9–11), and upregulation of inflammatory cytokines. Due to the clinical relevance of APAP toxicity, it is important to understand which immune system components and the cell signaling mechanisms are involved in APAP induced inflammation.

To control excessive cytokine effects, the cytokine signal is negatively regulated by a number of proteins, including protein inhibitor of activated STAT (PIAS; a signal transducer and activator of transcription), protein tyrosine phosphatases, and suppressors of cytokine signaling (SOCS) (12, 13). The family of SOCS proteins was originally described as comprising feedback inhibitors of cytokine-induced JAK/STAT signaling and consists of eight members, SOCS1-7 and cytokine-inducible Src homology 2 (SH2)-containing protein (CIS) (14). SOCS2 was originally described as a feedback inhibitor of the growth hormone (GH)/insulin-like growth factor axis (15). SOCS2 regulates the protein levels of other members, such SOCS1 and SOCS3, potentiating the signaling regulated by these SOCS proteins (16, 17).

Recently, SOCS2 has been recognized as a modulator of the immune system in different experimental models, playing several immune and oxidative stress regulatory functions, notably during infections (18–20). The absence of SOCS2 is related to unbalanced inflammatory response during *Toxoplasma gondii, Trypanosoma cruzi* and *Plasmodium berghei* ANKA infection (19–21). These studies demonstrated that SOCS2 is involved in a complex mechanism of balancing physiological functions of heart and brain by controlling the neurotrophic factors production and calcium handling, respectively, and being crucial for the generation/differentiation of the immune response, mainly by Th1, Th2, Th17, and T regulatory cells (18–21).

Here, the role of SOCS2 in the liver was assessed using a model of liver injury caused by acetaminophen overdose. In the absence of SOCS2, hepatic necrosis caused by APAP lead to increased through immune responses such as neutrophil recruitment, and cytokine and ROS generation. The findings suggest that SOCS2 plays a biologically important role in restraining deleterious immune responses in the liver upon APAP treatment. Our work offers insights into the signaling mechanisms involved in APAP-induced liver injury, and suggests new therapeutic targets to this important clinical problem.

## Materials and Methods

### Mice

Wild-type (WT) C57BL/6J male mice (8–10 weeks old) were obtained from the Centro de Bioterismo, Universidade Federal de Minas Gerais (UFMG), Minas Gerais, Brazil. SOCS2 knockout mice (SOCS2^−/−^) (8–10 weeks old) (15) were a kind gift from Dr. Warren S. Alexander (the Walter and Eliza Hall Institute of Medical Research, Australia). The study was carried out in strict accordance with Brazilian guidelines on animal work, and recommendations in the Guide for the Care and Use of Laboratory Animals of the NIH. All experiments and procedures were approved by the UFMG animal ethics committee (CETEA/UFMG, protocol 331/2015).

### Experimental Design for Drug-Induced Liver Injury Model

For the *in vivo* experiments, APAP was orally administered (600 mg/kg; Sigma-Aldrich, St. Louis, Missouri, USA) after 15 h of fasting. Control mice received warm sterile saline as a vehicle. In the survival experiments, mice were observed for 48 h. For the subsequent experiments, mice were anesthetized with a mixture of ketamine and xylazine (60 mg/kg and 15 mg/kg, respectively) after 2, 6, and 12 h of treatment and blood was obtained from the cava vein for evaluation of serum, and liver harvested for analysis. Intraperitoneal (i.p.) catalase (Sigma-Aldrich) was administrated at 5,000 U/kg 12 h before APAP, and in the moment of APAP challenge. In these experiments, mice were euthanized 2 h after APAP treatment.

### Biochemical Assays

Alanine aminotransferase (ALT) activity was estimated in serum using a kinetic assay kit (Bioclin, Brazil). The test is based on the consumption of pyruvate, formed in the presence of ALT in the serum sample. Consumption is proportional to the presence of ALT in the sample, and the result was measured in at 340 nm. Fragments from liver were collected to measure the reduced glutathione levels (GSH) (22) and myeloperoxidase (MPO) activity (11). The GSH quantification assay was performed in the liver (22). Samples were disrupted with a homogenizer and trichloroacetic acid, and centrifuged. The supernatant was incubated with 5,5′-dithiobis(2-nitrobenzoic acid) (0.25 M in methanol + Tris-HCl 1:3), and immediately measured at 415 nm. For determination of MPO activity, the assay included 25 μl of 3,3′,5,5′ tetramethylbenzidine (Sigma) in PBS (pH 5.4) as the color reagent. The number of neutrophils in each sample was calculated with reference to a standard curve of the number of neutrophils obtained from the peritoneal cavity of 5% casein–treated mice processed in the same manner, with results in the liver tissue expressed as the relative number of neutrophils per milligram of tissue wet weight.

### *In vivo* Mice Imaging

Liver confocal intravital microscopy was performed as described (23). Sytox Green (100 μL/mouse, 50 μM, Invitrogen, Carlsbad, CA, USA) and PE-conjugated anti-GR1 (4 μg/mouse; 40 μg/ml, eBioscience, San Diego, CA, USA) were injected intravenous (i.v.) 10 min before confocal microscopy imaging (Nikon, ECLIPSE 50i). Liver necrosis and neutrophil quantifications were performed using Volocity software (PerkinElmer).

### Histopathology

Liver samples from euthanized mice were obtained and processed for histopathological evaluation. Samples were fixed in 10% buffered formalin for 24 h and embedded in paraffin for tissue sectioning (5 μm thickness). The sections were stained with hematoxylin and eosin (H&E) and evaluated under a microscope (BX53, Olympus Latin America Inc.) adapted to a microcamera (Q-Color3, Olympus Latin America Inc., SP, Brazil). The histopathological score was adapted from Costa et al. (24), and evaluated hepatocytes degeneration and necrosis, inflammatory cell infiltration, and hemorrhage. A five-point score (0, absent; 1, minimal; 2, slight; 3, moderate; 4, marked; and 5, severe) was added to each parameter of analysis. The overall score was taken into account, and the results were plotted as the mean value of tissue damage for each mouse. The evaluations were performed in a blinded manner by a previously trained investigator.

### mRNA Expression

Quantification of mRNA expression was performed by real-time PCR (qPCR) analysis. Total RNA liver was isolated using RNeasy® Mini Kit (QIAGEN, Hilden, Germany). cDNA was generated by SuperScript III reverse transcriptase (Invitrogen), and amplified by SYBR green PCR Master Mix (Applied Biosystems, Foster City, CA, USA). The relative differences in expression between groups were expressed using threshold cycle (Ct) values generated by the ABI Prism 7500 Fast Sequence Detection System SDS (Life Technologies, Carlsbad, CA, USA). All genes evaluated were first normalized to GAPDH, and then expressed relative to a control arbitrarily set as 1.0. Calculations were performed using the 2^∧^(–ddCt) formula. The following primer sequences were used: TNF-α (forward 5′ACGGCATGGATCTCAAAGAC3′, reverse 5′AGATAGCAAATCGGCTGACG3′), IL-6 (forward 5′TTCCATCCAGTTGCCTTCTTG3′, reverse 5′TTGGGAGTGGTATCCTCTGTGA3′), IL-10 (forward 5′GCTCTTACTGACTGGCATGAG3′, reverse 5′CGCAGCTCTAGGAGCATGTG3′), CXCL-1 (forward 5′TGTCCCCAAGTAACGGAGAAA3′, reverse 5′TGTCAGAAGCCAGCGTTCAC3′), TGF-β (forward 5′GAGGTCACCCGCGTGCTA3′, reverse 5′TGTGTGAGATGTCTTTGGTTTTCTC3′) and GAPDH (forward 5′ACGGCCGCATCTTCTTGTGCA3′, reverse 5′CGGCCAAATCCGTTCACACCGA3′).

### Western Blot

Liver tissues were homogenized with lysis buffer, and lysates were centrifuged at 15,000 g for 15 min. Proteins (80 μg) were separated on a 12% SDS-polyacrylamide gel, and then transferred to a nitrocelulose membrane (Millipore, Billerica, MA, USA). After blocking in 5% (w/v) non-fat dried milk dissolved in Tris-buffered saline (10 mM Tris, 150 mM NaCl and 0.05% Tween-20, pH 7.8), the membrane was incubated with specific primary antibody against SOCS2 (1:1,000, Cell Signaling, Danvers, MA, USA), or β-actin (1:5,000, Sigma–Aldrich) for 2 h at 23°C. The blots were then incubated in the corresponding horseradish peroxidase-conjugated immunoglobulin g (Cell Signaling). The immunocomplex was detected by the ECL detection system (GE Healthcare, Little Chalfont, United Kingdom). The relative density of the protein bands was quantified by densitometry using Image J (U. S. National Institutes of Health, Bethesda, Maryland, USA). The density of each band was normalized by background and β-actin.

### Primary Murine Hepatocytes Culture

Primary hepatocytes purification was performed as described previously (25). Briefly, mice were anesthetized and submitted to liver perfusion with collagenase (Sigma-Aldrich, C2139) through the portal vein. After the perfusion, the liver was dissociated in Williams' E medium, and the material was filtered through a 40 μm sterile nylon mesh. After centrifugation, cell viability was determined by trypan blue dye, and the hepatocytes were plated in Williams' E medium supplemented with 10% fetal bovine serum and incubated at 37°C in 5% CO2 humidified incubator. Hepatocytes from WT and SOCS2^−/−^ were treated with different stimulus: APAP at 5 mM dissolved in 1% dimethyl sulfoxide (DMSO), hydrogen peroxide (Sigma-Aldrich) at 250 mM, catalase at 60 U/mL, IL-6 (R&D Systems) at 100 ng/mL, and TNF-α (R&D Systems) at 100 ng/mL. After specific times, hepatocytes viability was assessed by the release of lactate dehydrogenase (LDH) in supernatant using a LDH kinetic assay kit (Bioclin, Brazil).

### Reactive Oxygen Species (ROS) Production

Primary hepatocytes treated with 5 mM APAP were incubated with 25 μM of 2′,7′-dichlorodihydrofluorescein diacetate (H2DCF-DA; Invitrogen) for 30 min to assess the intracellular ROS generation. The samples were quantified by fluorimetry. Data were expressed as fold change in arbitrary units of fluorescence.

### IkBα, p-IkBα, and GAPDH Expression in Primary Hepatocytes

After incubation with 5 mM of APAP (15, 30, 45, and 90 min), protein suspensions derived from primary hepatocytes were collected for western blot analysis. Proteins (30 μg) were loaded and electrophoresed on a 12% SDS-polyacrylamide gel as described above. The activation of MAPK proteins was evaluated using specific primary antibodies to total IkBα, p-IkBα, and GAPDH (1:1,000, Cell Signaling).

### Immunofluorescence

Hepatocytes were fixed, permeabilized, blocked and incubated with primary NFkb antibody (1:100; Cell Signaling), followed by propidium iodide (PI, 30 μM; Sigma-Aldrich) for 30 min. Images were obtained using confocal microscopy (Nikon, ECLIPSE 50i). The linear fluorescence profile was obtained using Volocity software (PerkinElmer).

### Statistical Analysis

Results are shown as the mean ± standard error (SEM). Differences for variables normally distributed were compared by using Student's *t*-test (unpaired) or analysis of variance (ANOVA). Bonferroni post-test was used as needed for multiple comparisons. The significance level was set at the level of *p* < 0.05. Statistical analyses were performed using Prism 5 software (GraphPad, La Jolla, CA, USA).

## Results

### APAP challenge Causes Downregulation of SOCS2 Expression in the Liver of WT Mice

**Figure 1:**
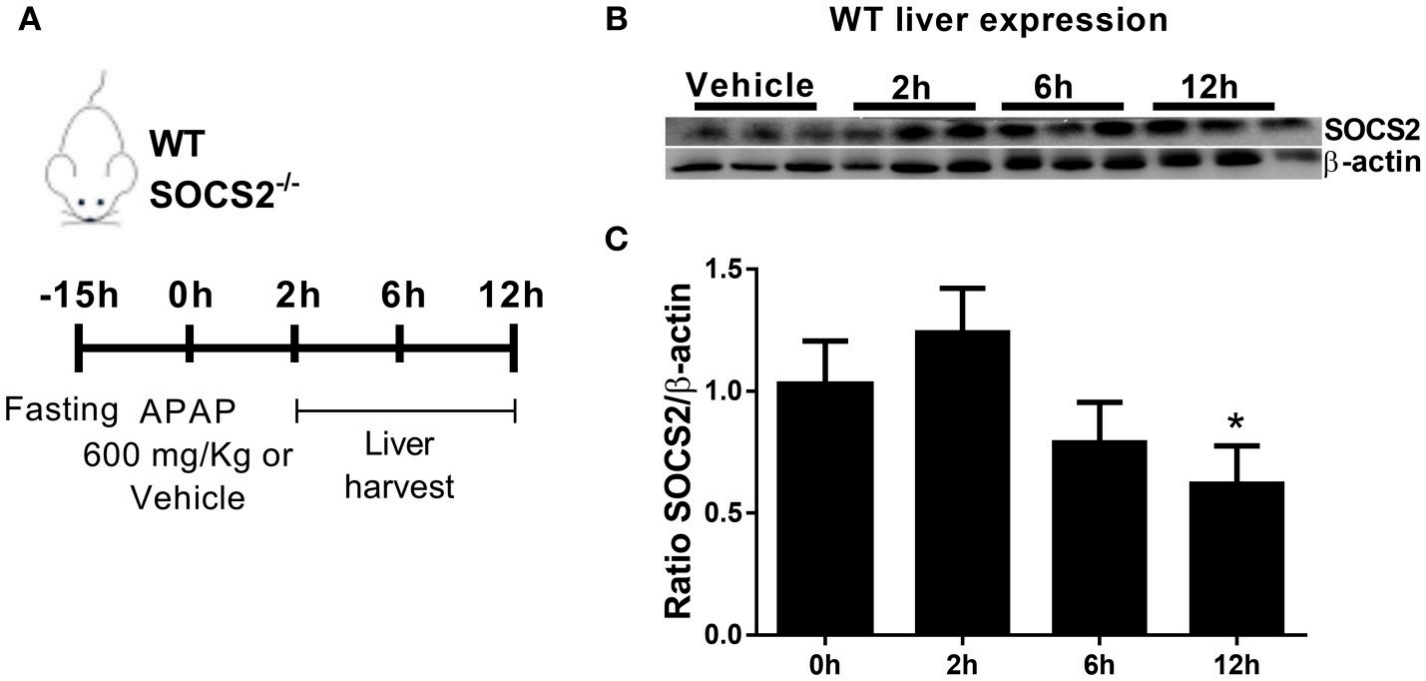
Expression of SOCS2 in the liver decreased after administration of APAP. **(A)** *In vivo* treatment schedule with APAP. After 15 h of fasting, WT and SOCS2^−/−^ animals receive 600 mg/kg of APAP or saline as a control. After 2, 6, and 12 h, liver and serum were collected for analysis. **(B)** Representative western blot of SOCS2 and β-actin expression in the liver of WT animals (*n* = 3) after 0 (control), 2, 6, and 12 h of APAP administration (600 mg/kg). **(C)** Densitometry referring to **(B)** were the expression of SOCS2 was normalized with respect to the expression of the endogenous β-actin gene. The figure shows the means of three independent experiments (*n* = 3 per group) ± SEM. ^*^p < 0.05 (statistical significance performed by one-way ANOVA, with Tukey post-test).

In order to examine a putative role for SOCS2 in the modulation of APAP toxicity, we determined SOCS2 expression levels in the liver of WT mice treated with 600 mg/kg of APAP Figure 1A. SOCS2 was upregulated 2 h after APAP treatment, and then decreased to below background levels after 12 h of treatment (Figures 1B,C).

### APAP-Induced Mortality and Liver Necrosis Are Increased in SOCS2^−/−^ Mice

**Figure 2:**
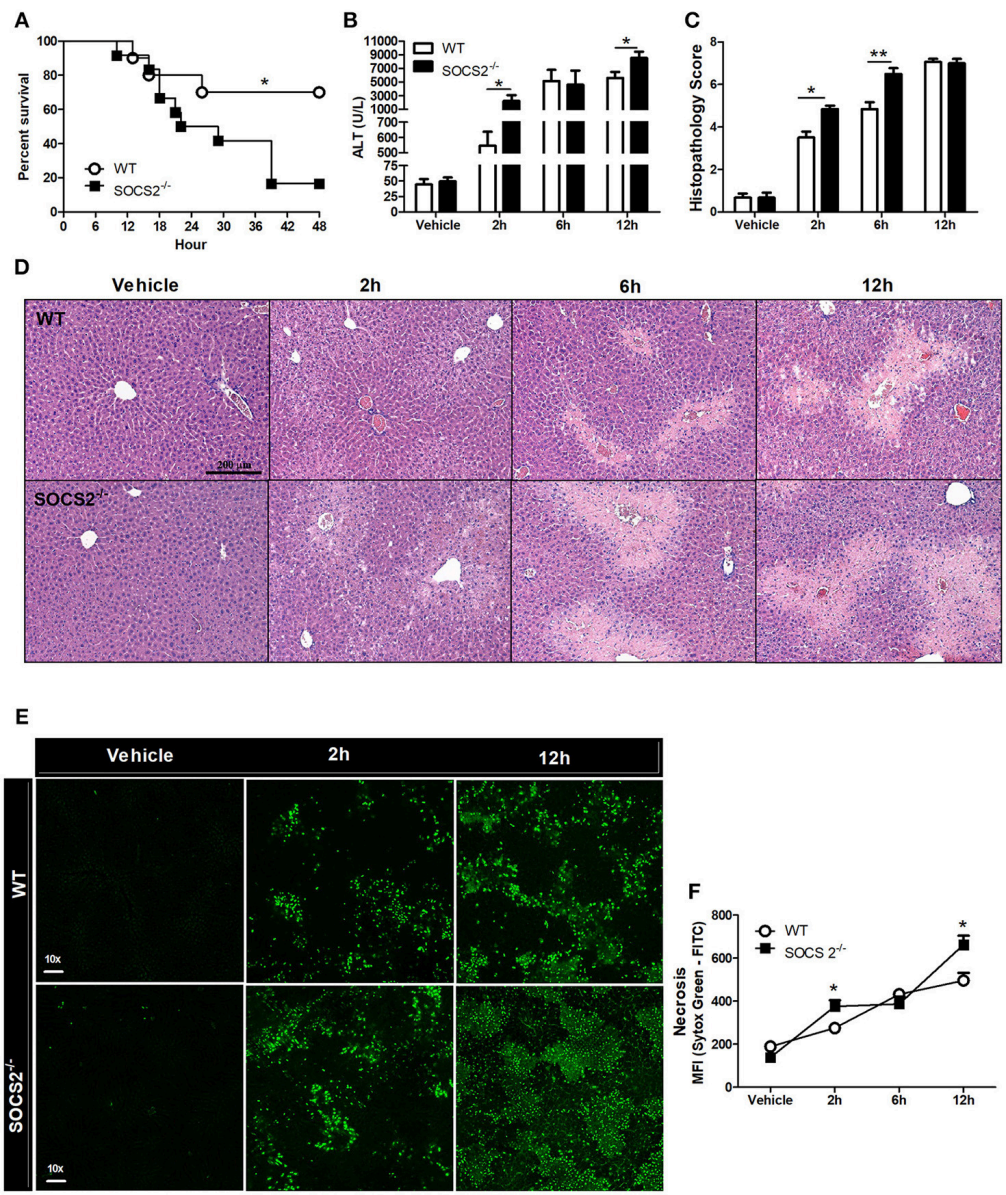
Deficiency of SOCS2 promoted increased lethality and necrosis in mice after APAP treatment. **(A)** Percentage of death from WT and SOCS2^−/−^ mice (*n* = 12), observed for 48 h after administration of APAP (600 mg/kg). ^*^p < 0.05 (statistical significance performed by the log-rank test). **(B)** Release of ALT in the serum (*n* = 4) and **(C)** histopathological score calculated based on the parameters necrosis, degeneration, hemorrhage and inflammatory infiltrate at the time indicated in the figures. Values used for evaluation were: 0, absent; 1, minimum; 2, light; 3, moderate; 4, striking; 5, severe. **(D)** Representative image of hepatic histology with H&E staining (20x magnification). **(E)** Stained fluorescence of the Sytox Green Marker (green); Intensity of fluorescence (MFI). **(F)** Representative image of intravital confocal microscopy (*n* = 3) in which necrosis was marked by Sytox Green (increase of 60x). Data were expressed as the mean ± SEM. ^*^p < 0.05; ^**^p < 0.01 (statistical significance performed by Student's *T*-test).

To assess SOCS2 function, the survival of APAP-treated WT and SOCS2^−/−^ mice was determined. APAP challenge of WT mice was accompanied by ~30% lethality rate at 48 h. Lethality rates for SOCS2^−/−^ mice were significantly higher (Figure 2A). The reduced survival rate in SOCS2^−/−^ mice can be explained by increased ALT levels detected in the serum, a specific marker of liver injury, at 2 and 12 h after APAP administration (Figure 2B). The analysis of tissue sections demonstrated greater areas of necrosis in SOCS2^−/−^ mice at 2 and 6 h after challenge, an effect that was clearly observed in the histophatological scores, when compared with WT counterparts (Figures 2C,D). In addition, APAP-induced liver injury was associated with a marked increase in tissue Sytox Green staining (demonstrating DNA release by necrotic cells) in the liver of SOCS2^−/−^ compared to WT mice (Figures 2E,F).

### SOCS2 Controls Inflammation During APAP-Induced Injury

**Figure 3:**
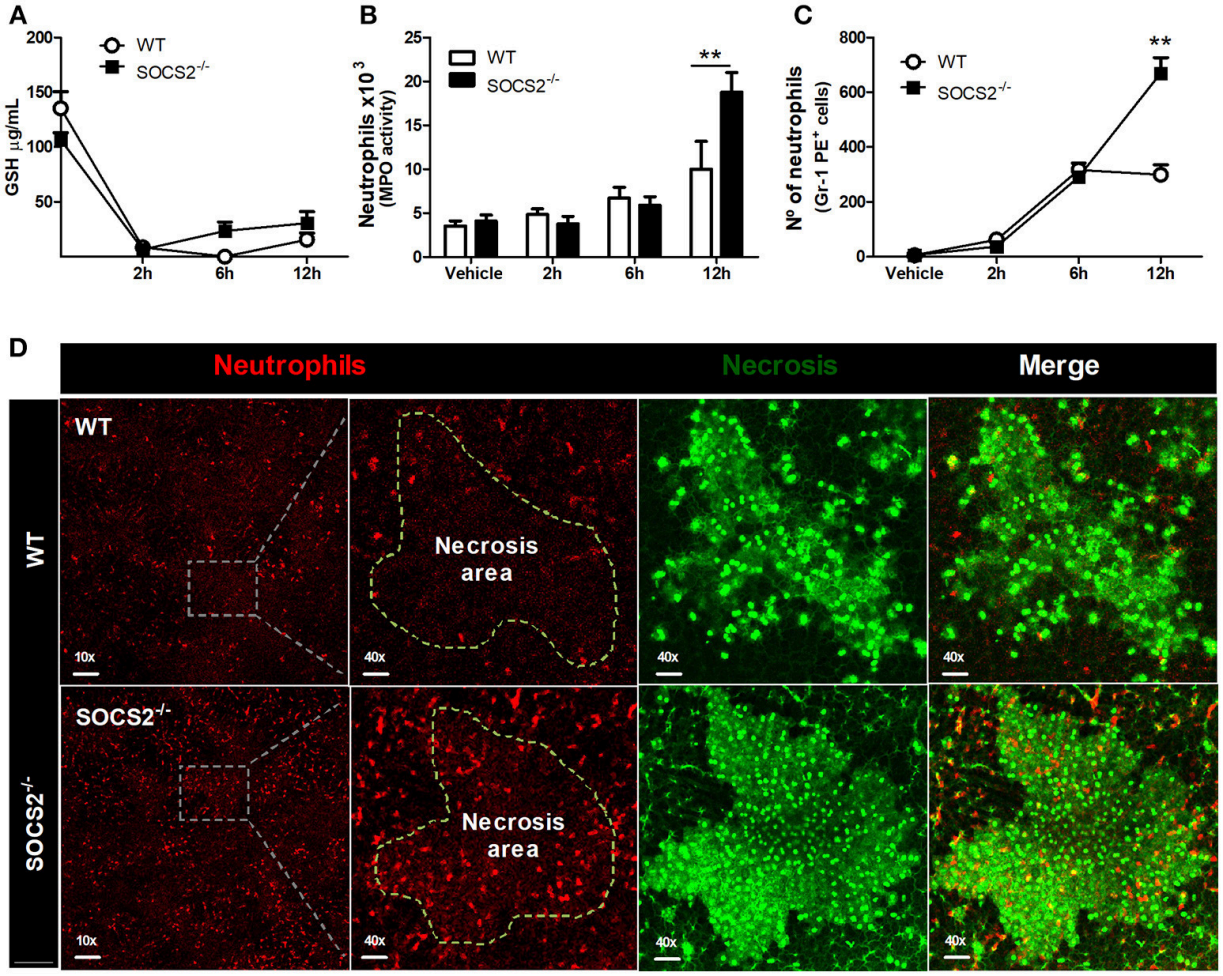
Deficiency of SOCS2 resulted in higher necrosis, without alteration of metabolic factors in the liver after APAP treatment. Analyses were performed in the liver of WT animals (*n* = 4) and SOCS2^−/−^ (*n* = 4) after treatment with APAP (600 mg/kg). **(A)** Measurement of GSH in the liver using a colorimetric reaction with DNTB. Data represent the mean ± SEM. **(B)** The number of neutrophils estimated by determination of MPO activity as described in the material and methods. **(C)** Intravital confocal/quantification of neutrophils (Gr1-PE^+^ cells) using the Volocity program 6.3. **(D)** Intravital confocal microscopy shows the staining with antibody Gr1-PE + (red)—neutrophils—within the hepatic necrotic area (marked with Sytox Green—green) 12 h after treatment. The merge figures represent the overlap of the neutrophils and necrosis images (40x magnification). Data represent mean ± SEM. ^**^p < 0.01 (statistical significance performed by Student's *T*-test).

**Figure 4:**
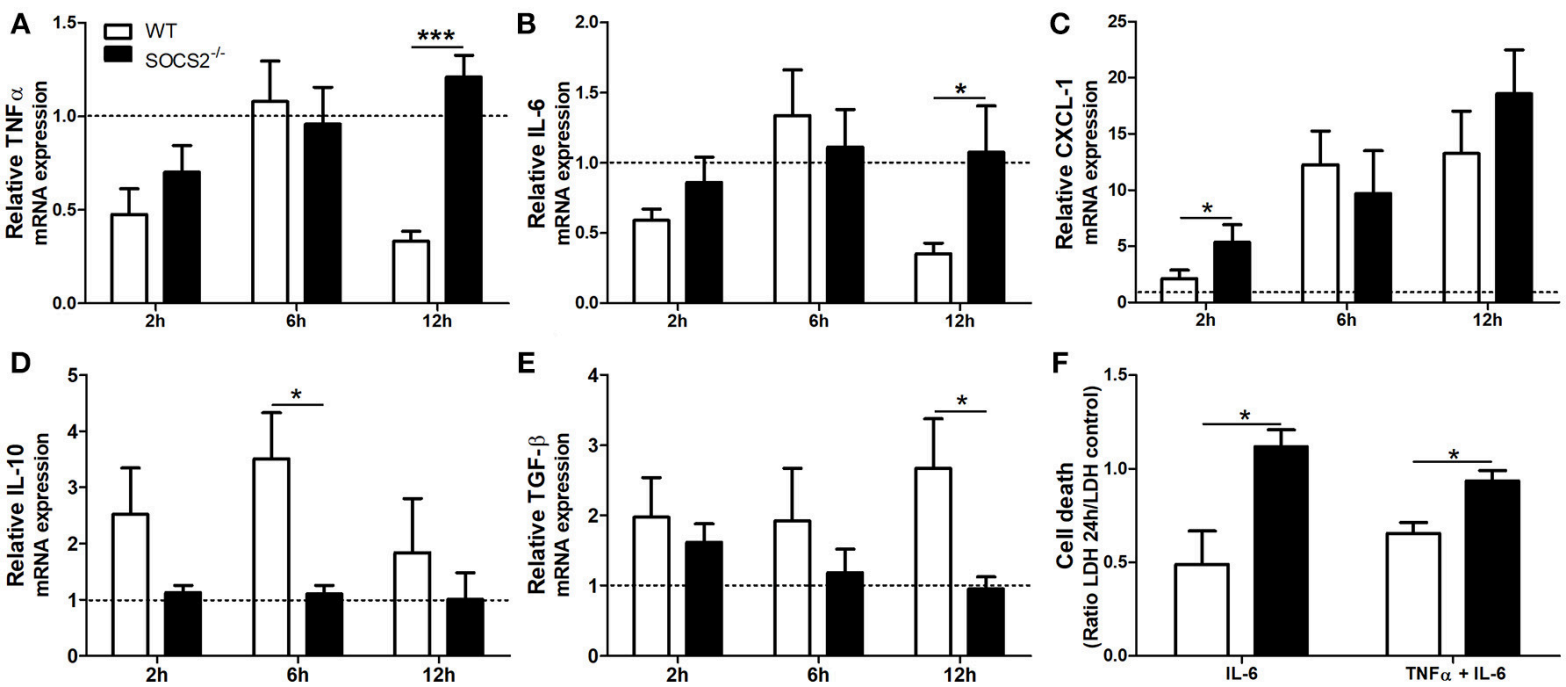
Deficiency of SOCS2 lead to different expression of inflammatory genes in mice livers after APAP overdose, and enhanced hepatocyte death after stimulus with pro-inflammatory cytokine *in vitro*. WT animals (*n* = 4 per group) and SOCS2^−/−^ (*n* = 4 per group) were treated with APAP (600 mg/kg) and euthanized after 2, 6, and 12 h. Livers were harvested for qPCR analysis of genes related to cytokines and chemokine. The results were normalized with the endogenous GAPDH gene, and expressed in relation to the vehicle (saline) treated control. The figures show the means of three independent experiments (*n* = 4 per group) ± SEM. ^*^p < 0.05; ^***^p < 0.001 (statistical significance performed by one-way ANOVA, with Tukey post-test). **(A)** TNF-α, **(B)** IL-6, **(C)** CXCL-1, **(D)** IL-10 e **(E)** TGF-β relative expression. **(F)** Hepatocytes death measured by LDH (lactate dehydrogenase) release after incubation with stimulus [IL-6 (100 ng/mL) alone or in combination with TNF-α (100 ng/mL)]. Cell death was represented as a ratio of LDH release by hepatocytes in the presence of cytokines relative to LDH release by vehicle treated cells. Data are represented as the mean ± SEM. ^*^p < 0.05 (statistical significance was performed by Student's *T*-test).

First, because conditions that interfere with metabolism and metabolic activation can alter the hepatotoxicity of the APAP, we evaluated if the increased metabolism of the drug was responsible for the higher injury detected in SOCS2^−/−^ mice. As shown in Figure 3A, depletion of GSH was similar between WT and SOCS2^−/−^ at the basal levels and during all time of kinetics analyzed after APAP treatment. Next we investigated the role of this protein in the cellular recruitment. MPO activity, an indirect marker of neutrophil presence in tissue, was increased in the liver of SOCS2^−/−^ mice at 12 h after challenge when compared with WT (Figure 3B). Intravital analyses of neuthophils stained with Gr1 PE^+^ confirmed the higher infiltration of these cells in the liver of KO mice, mainly in the necrotic area, at 12 h after APAP challenge when compared with WT (Figures 3C,D). In addition, SOCS2 deficient mice treated with APAP also displayed an increased expression of TNF-α, IL-6, and CXCL-1 and decreased expression of IL-10 and TGF-β hepatic gene, when compared with WT counterparts (Figures 4A–E). Because cytokines such as TNF-α and IL-6 are associated with protein oxidation and membrane damage caused by ROS and hepatocyte susceptibility during APAP overdose *in vivo*, we purified WT and SOCS2^−/−^ hepatocytes and *in vitro* stimulated them with IL-6 and TNF-α in order to determine their susceptibility in this environment. Compared to WT cells, SOCS2 deficient hepatocytes were more susceptible to the presence of pro-inflammatory cytokines IL-6 and TNF-α *in vitro*, as shown by higher cell death (Figure 4F).

### SOCS2 Regulates the Expression of p-IkBα and p-NF-κB (p-p65) in Hepatocytes Upon APAP Treatment

**Figure 5:**
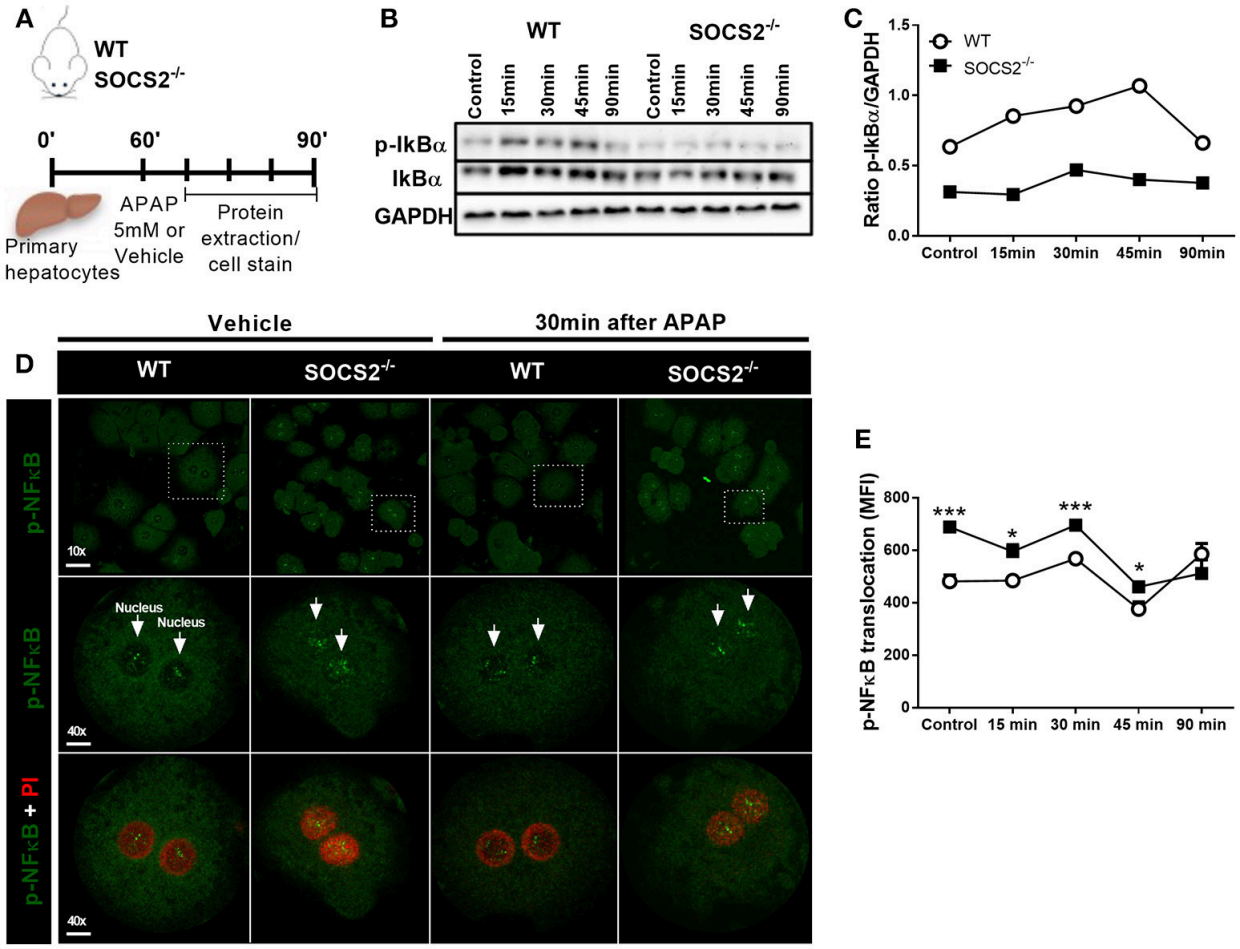
Modulation of IKBα/NF-κB expression in hepatocytes by SOCS2 upon APAP incubation. **(A)** Schematic representation of the primary hepatocytes culture (2 × 10^5^ cells/well) of WT and SOCS2^−/−^ mice, isolated and cultured *in vitro* with APAP (5 mM), during the kinetics of 0, 15, 30, 45, and 90 min. After each time, cells were lysed and the proteins collected for western blot analyzes. **(B)** Western blot analysis of total IkBα and p-IkBα (37 kDa) and endogenous GAPDH control (37 kDa). **(C)** Densitometry corresponding to p-IkBα western blot normalized to total IkBα levels. **(D)** Representative image of *in vitro* NF-κB translocation in primary hepatocytes isolated from WT and SOCS2^−/−^ mice 30 min after incubation with APAP (5 mM) or vehicle. Hepatocytes were labeled with FITC (green) for the NF-kB protein p-p65, and with PI (red) for the nucleus. **(E)** Quantification of p-p65 translocation to hepatocyte nucleus at 0, 15, 30, 45 and 90 min after incubation with APAP (5 mM). The mean fluorescence intensity was determined by the Volocity 6.3 software. Data were expressed as the mean ± SEM (*n* = 5). ^*^p < 0.05; ^***^p < 0.001 (statistical significance performed by Student's *T*-test).

To investigate the hypotheses that SOCS2 mediates counter-regulatory activity in hepatocytes, an *in vitro* model of primary hepatocytes isolated from WT and SOCS2^−/−^ mice was used. The expression of IkBα **and p-**IkBα was analyzed at 15, 30, 45, and 90 min after APAP stimulus (Figure 5A). The pIkBα/IkBα ratio, one of the triggers for NF-κB activation, was diminished after APAP stimulus in KO cells compared to WT cells (Figures 5B,C). Accordingly, the translocation and consequent activation of p-NF-κB is higher in SOCS2^−/−^ hepatocytes at basal levels up to 45 min after APAP incubation when compared with WT cells (Figures 5D,E).

### APAP-Induced Oxidative Stress Was Increased in SOCS2^−/−^ Hepatocytes

**Figure 6:**
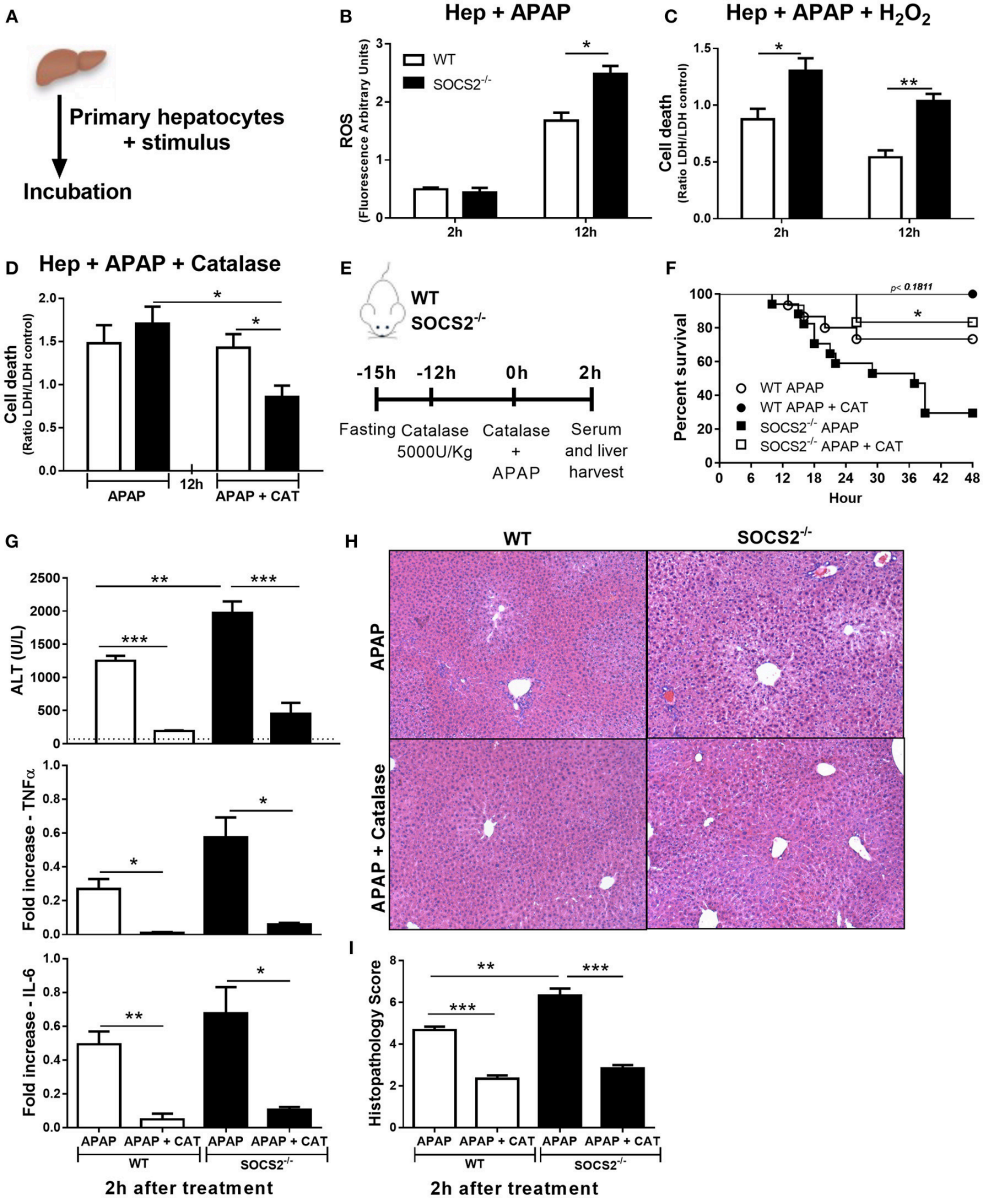
Role of SOCS2 in the regulation of oxidative stress and necrosis levels during APAP treatment. **(A)** Schematic representation of *in vitro* experiments performed on items **(B–D)**. 2 × 10^5^ primary hepatocytes/well were incubated with different stimuli. **(B)** The ROS probe (H2DCF-DA, 25 μM) was added to the primary hepatocytes, and after 2 and 12 h the arbitrary fluorescence units were detected and normalized relative to the vehicle-treated control. **(C)** Cell death of the primary hepatocytes cultured with APAP (5 mM) and H_2_O_2_ (250 mM). Cell viability was assessed after 2 and 12 h by the release of the LDH in the culture supernatant, and the result was expressed in relation to the vehicle treated control. **(D)** Effects of treatment with 60 U/mL of catalase (CAT) to reduce cell death after 12 h treatment of hepatocytes with APAP (5 mM). Data represent the mean ± SEM. ^*^p < 0.05; ^**^p < 0.01 (statistical significance was performed by Student's *T*-test). **(E)** Representative scheme of *in vivo* experiments to evaluate the action of catalase on hepatic necrosis. WT and SOCS2^−/−^ mice were treated with 5000 U/kg i.p. of CAT 12 h (*n* = 6) or not (*n* = 12) before APAP treatment. At the time of administration of 600 mg/kg APAP, animals were challenged with the same dose of catalase given above. **(F)** Percentage of death from WT and SOCS2^−/−^ mice, observed for 48 h after treatments. ^*^p < 0.05 (statistical significance performed by the log-rank test). **(G)** After 2 h of treatments, the animals were sacrificed and the serum and liver collected for analysis. Measurement of serum ALT release from mice after APAP alone and with catalase treatments. The liver was collected for qPCR analysis of genes related to TNF-α and IL-6. The results were normalized with the endogenous GAPDH gene, and expressed in relation to the vehicle (saline) treated control. **(H)** Representative images of hepatic histology. The arrows indicate the areas of centrolobular necrosis. **(I)** Histopathological score of the liver from these animals evaluated: necrosis, degeneration, hemorrhage and inflammatory infiltrate. Values used for evaluation were: 0, absent; 1, minimum; 2, light; 3, moderate; 4, striking; 5, severe. Data represent the mean ± SEM. ^**^p < 0.01; ^***^p < 0.001 (statistical significance was performed by Student's *T*-test).

ROS is an important cytotoxic mediator produced by liver during APAP intoxication. Thus, we evaluated if SOCS2 modulates the levels of ROS formation in hepatocytes upon APAP stimulus Figure 6A. SOCS2 deficient hepatocytes produced increased levels of ROS 12 h after APAP stimulus compared to WT cells (Figure 6B). In addition, the deficiency of SOCS2 resulted in greater APAP sensitivity to H_2_O_2_ enhancing cell death relative to WT (Figure 6C).

### Catalase Treatment Decreased the Exacerbated Oxidative Stress Production Found in APAP-Treated SOCS2^−/−^ Mice

Catalase is the major enzyme responsible for H_2_O_2_ metabolism. The addition of catalase decreased the toxicity of APAP in SOCS2^−/−^, but not in WT hepatocytes *in vitro* (Figure 6D). The efficacy of catalase treatment was tested *in vivo* in mice pre-treated with catalase 12 h before and at the time of APAP challenge (Figure 6E). Treatment with catalase reduced dramatically the SOCS2^−/−^ mice mortality (Figure 6F), and the ALT levels (Figure 6G), and TNF-α and IL6 expression (Figure 6G) in liver of both WT and SOCS2^−/−^ mice. Although SOCS2^−/−^ mice still presented more necrosis in the liver compared with WT, the reduction in this transaminase reaches the same level found in WT mice (Figure 6G). The reduction of necrosis around the centrilobular area was shown in the histopathological sections of treated mice subjected to APAP injury (Figures 6H,I).

## Discussion

Because APAP is present in many formulations, alone or in combination with other drugs, there is an increasing concern of excessive dosage causing liver damage. In addition, alcohol use, malnutrition, and other factors can predispose one to APAP induced hepatotoxicity, even when administered in recommended or low doses (1). The development of these idiosyncratic reactions involves genetic determinants and may be drug-specific (26), as well as having critical involvement of immune mechanisms (27). SOCS2 has important roles in the immune system, especially in autoimmunity and infection (19–21, 28). It is important to understand how key molecules in balancing of the immune system and physiological functions, such as SOCS2, can influence the response to APAP intoxication. Here, we demonstrated that after APAP overdose SOCS2 has roles in: (i) controlling liver necrosis; (ii) decreasing neutrophil recruitment; (iii) contributing to the preservation of an anti-inflammatory profile in hepatocytes, and (iv) helping to regulate oxidative stress.

SOCS2 is expressed in many organs, and was shown to balance metabolic and restorative requirements during liver regeneration after partial hepatectomy (29). We demonstrated here that upon *in vivo* APAP treatment, WT mice displayed a significant decrease in SOCS2 expression in the liver. Moreover, SOCS2 deficient mice treated with APAP presented elevated mortality when compared with WT, demonstrating that the presence of SOCS2 contributes to the survival of the animals in this model. To confirm these observations, we performed *in vivo* and *in situ* analysis of liver from WT and SOCS2-deficient mice. Deficiency of SOCS2 resulted in higher hepatic necrosis associated with increased in ALT levels, which can be observed as early as 2 h after administration. At 6 h, this difference is the same, and becomes higher in KO animals 12 h after intoxication when compared with WT. These observations were confirmed by confocal microscopy. Histology showed a slightly different profile, with greater necrosis in SOCS2^−/−^ mice 2 and 6 h after APAP administration. The differences may be due to the fact that in addition to necrosis, histology also evaluates degeneration, cellular infiltration, and hemorrhage. Despite this, histology confirmed that the type of lesion generated by the APAP is the same in the WT and SOCS2^−/−^ groups of animals: necrosis around the centrilobular area and in the pericentral hepatocytes. SOCS2 has heterogeneous expression in hepatocytes, being more expressed in the hepatocytes of the pericentral areas (30). The lack of SOCS2 in the cells in thedeath area seems to influence the degree and extent of necrosis. Taken together, the aforementioned observations suggests that there may be a differential initial mechanism that causes a more severe necrosis in SOCS2^−/−^ mice, and another that occurs later, making these animals more inflamed, and consequently causing greater lethality compared to WT mice.

The difference between reduction in hepatic expression of SOCS2 protein after 12 h of APAP overdose found here, and greater expression of SOCS2 mRNA in the liver after 6 h found by another study (31) may be justified by the fact that members of the SOCS family have marked post-transcriptional regulation, such as the inter-regulation that occurs between them. Additionally, SOCS2 acts as a negative regulator of other members of the family, such as SOCS1 and 3, and is therefore more expressed in late phases. SOCS3 has been described as an important factor in the increased liver damage caused by APAP, mainly by increasing the production of inflammatory cytokines such as TNF-α and IFN-γ (32). Therefore, a possible cause for higher necrosis found in the liver of the SOCS2^−/−^ animals could be the amplification of the process discussed above, mainly due to the greater activation of the immune system.

Considering that neutrophils are recruited into the inflammation area in response to chemokines and mitochondrial products, amplifying liver injury (11), and that SOCS2 modulates chemokine production and the migration of neutrophil-producing TNF-α to the peritoneal cavity upon LPS challenge, we asked whether chemokine production and migration of neutrophils to the liver was under the control of SOCS2 upon *in vivo* APAP treatment. There was a considerable increase in the number of neutrophils in both WT and SOCS2^−/−^ groups, but SOCS2^−/−^ animals presented increased recruitment of neutrophils 12 h after administration of APAP compared to WT. Furthermore, the neutrophils localized within the area of necrosis, corroborating the behavior of patrolling in this location (33). We also demonstrated higher expression of CXCL-1 chemokine in SOCS2^−/−^ animals. The CXCR-2 receptor binding chemokines have important roles in neutrophil recruitment to inflamed liver (11). The results suggest that *in vivo* regulation of APAP-induced chemokine production, and consequently neutrophil migration, is at least partially controlled by SOCS2.

We next asked whether SOCS2 plays a broader role in the intracellular regulatory pathways triggered by APAP. The IL-6 cytokine has a proinflammatory role and is more expressed in the liver of SOCS2 deficient animals. Elevated levels of IL-6 in the liver are associated with acute and chronic diseases (34). IL-6 binds directly to the receptor in the membrane of hepatocytes, activating protein expression via the JAK-STAT3 and NF-κB pathways (35). Because growth hormone and IL-6 are more abundant in the absence of SOCS2, it is possible that they are associated with higher activation of NF-κB found in this group of animals.

The lower activation of IkBα in the SOCS2^−/−^ animals could be related to the higher expression of NF-κB (p65 subunit, also called RelA) found in these animals. In the canonical pathway of NF-κB activation, phosphorylation of IkBα marks it for proteasome degradation, releasing NF-κB to the nucleus. Hepatocytes from SOCS2^−/−^ mice showed increased activation of NF-κB indicating that low levels of IkBα activation could be its degradation before stimulus. Although IKBα activation is the most studied and related to NF-κB activation, it is important to mention that that IkB has several isoforms, such as IkBβ and IkBε. It has also been shown that SOCS2^−/−^ mice had increased basal NF-κB expression in another hepatic model, corroborating our results (36).

In most cases, NF-κB controls the expression of target genes related to cell survival, however, in some instances it can promote cell death (37). ROS are able to modulate the NF-κB response, and ROS targets can attenuate the response promoting survival. In our model, higher NF-κB expression correlated with increased ROS production in SOCS2-deficient hepatocytes. ROS can interact with NF-κB at various stages of its signaling. ROS may stimulate NF-κB signaling by activating IKK, and downstream pathways, phosphorylation of IkBα, prompting its degradation, or phosphorylation of p65, leading to increased NF-κB translocation and binding to DNA. NF-κB alters ROS levels primarily by increasing the expression of antioxidant enzymes. However, this effect seems to be contrary to catalase. Schreiber et al. (38) suggested that catalase levels could decrease when there is canonical activation of NF-κB. Because SOCS2^−/−^ animals presented greater activation of NF-κB, we investigated if the addition of catalase to the system caused a decrease in the oxidative stress, and consequently the death of hepatocytes in the presence of APAP. Decreased necrosis and death in both WT and SOCS2^−/−^ mice that received catalase before and after APAP overdose supports this idea. SOCS2 deficient mice presented necrosis at the same level of WT animals, showing that oxidative stress can be reversed by the action of catalase 2 h after APAP intoxication. Treatment with catalase also reduced the expression of TNF-α and IL-6 in the liver of WT and SOCS2^−/−^ mice. Antioxidant treatment reduces phosphorylation of p65 at Ser276, which is necessary for its activity (39). Therefore, besides directly degrading ROS, catalase treatment may reduce NF-κB activity, which contribute to lower cell death found *in vitro* and *in vivo*. The catalase-induced reduction of ALT levels in SOCS2 ^−/−^ animals did not reach normal levels, but this may not be of clinical importance. Asymptomatic elevations of ALT 3 or more times above normal levels have been detected in chronic users of APAP within dosage limits. The clinical importance of these elevations during therapeutic use is unclear (40).

The present study revealed a clear phenotype in the absence of SOCS2 in model of hepatic inflammation generated by overdose of APAP. The higher lethality of these animals was due to the greater injury that occurred in the liver. APAP toxicity induced greater activation of NF-κB, which may have led to increase ROS production by hepatocytes. In the absence of SOCS2 the higher activation of these proteins may be related to higher production of ROS, which increases the initial injury (up to 2 h after treatment). The administration of catalase changed this profile, restoring the levels closer to those of WT animals. In addition, increased production of pro-inflammatory factors such as IL-6 and TNF-α, decreased production of IL-10 and TGF-β, and increased recruitment of neutrophils may amplify the lesion, which occurred 12 h after APAP intoxication. Further studies are required to understand the role of SOCS2 in the development of other adverse effects of APAP, such as kidney, pulmonary, endocrine, neurological, and neurodevelopmental toxicity. Collectively, our results suggested that the SOCS2 is a key regulator of the immune response in the liver after APAP treatment. Genetic polymorphisms in SOCS2, or failure in the induction of its expression may increase the susceptibility of hepatotoxicity caused by APAP.

## Author Contributions

RM-R, FM, and GM conceived experiments. RM-R, FM, and GM wrote the manuscript. RM-R, AC, PG, MA, RP, AB, and BD performed experiments. CQ-J performed histological experiments and analysis. FM, GM, and MT supervised and provided expertise and funding.

### Conflict of Interest Statement

The authors declare that the research was conducted in the absence of any commercial or financial relationships that could be construed as a potential conflict of interest.

## References

[1] Larson AM. Acetaminophen hepatotoxicity. Clin Liver Dis. (2007) 11:525–48. 10.1016/j.cld.2007.06.006 17723918

[2] Amar PJ Schiff ER. Acetaminophen safety and hepatotoxicity—where do we go from here? Expert Opin Drug Saf. (2007) 6:341–55. 10.1517/14740338.6.4.341 17688378

[3] Jaeschke H Williams CD Ramachandran A Bajt ML. Acetaminophen hepatotoxicity and repair: the role of sterile inflammation and innate immunity. Liver Int. (2012) 32:8–20. 10.1111/j.1478-3231.2011.02501.x 21745276 PMC3586825

[4] Nelson SD. Molecular mechanisms of the hepatotoxicity caused by acetaminophen. Semin Liver Dis. (1990) 10:267–78. 10.1055/s-2008-1040482 2281334

[5] Donnelly PJ Walker RM Racz WJ. Inhibition of mitochondrial respiration *in vivo* is an early event in acetaminophen-induced hepatotoxicity. Arch Toxicol. (1994) 68:110–8. 8179480 10.1007/s002040050043

[6] Meyers LL Beierschmitt WP Khairallah EA Cohen SD. Acetaminophen-induced inhibition of hepatic mitochondrial respiration in mice. Toxicol Appl Pharmacol. (1988) 93:378–87. 3368917 10.1016/0041-008x(88)90040-3

[7] Hanawa N Shinohara M Saberi B Gaarde WA Han D Kaplowitz N. Role of JNK translocation to mitochondria leading to inhibition of mitochondria bioenergetics in acetaminophen-induced liver injury. J Biol Chem. (2008) 283:13565–77. 10.1074/jbc.M708916200 18337250 PMC2376214

[8] Martin-Murphy BV Holt MP Ju C. The role of damage associated molecular pattern molecules in acetaminophen-induced liver injury in mice. Toxicol Lett. (2009) 192:387–94. 10.1016/j.toxlet.2009.11.016 19931603 PMC2822049

[9] Liu ZX Han D Gunawan B Kaplowitz N. Neutrophil depletion protects against murine acetaminophen hepatotoxicity. Hepatology (2006) 43:1220–30. 10.1002/hep.21175 16729305

[10] Liu ZX Govindarajan S Kaplowitz N. Innate immune system plays a critical role in determining the progression and severity of acetaminophen hepatotoxicity. Gastroenterology (2004) 127:1760–74. 10.1053/j.gastro.2004.08.053 15578514

[11] Marques PE Amaral SS Pires DA Nogueira LL Soriani FM Lima BH. Chemokines and mitochondrial products activate neutrophils to amplify organ injury during mouse acute liver failure. Hepatology (2012) 56:1971–82. 10.1002/hep.25801 22532075

[12] Rakesh K Agrawal DK. Controlling cytokine signaling by constitutive inhibitors. Biochem Pharmacol. (2005) 70:649–57. 10.1016/j.bcp.2005.04.042 15936728

[13] Flores-Morales A Greenhalgh CJ Norstedt G Rico-Bautista E. Negative regulation of Growth hormone receptor signaling. Mol Endocrinol. (2006) 20:241–53. 10.1210/me.2005-0170 16037128

[14] Starr R Willson TA Viney EM Murray LJ Rayner JR Jenkins BJ. A family of cytokine-inducible inhibitors of signalling. Nature (1997) 387:917–21. 10.1038/43206 9202125

[15] Metcalf D Greenhalgh CJ Viney E Willson TA Starr R Nicola NA. Gigantism in mice lacking suppressor of cytokine signalling-2. Nature (2000) 405:1069–73. 10.1038/35016611 10890450

[16] Piessevaux J Lavens D Montoye T Wauman J Catteeuw D Vandekerckhove J. Functional cross-modulation between SOCS proteins can stimulate cytokine signaling. J Biol Chem. (2006) 281:32953–66. 10.1074/jbc.M600776200 16956890

[17] Tannahill GM Elliott J Barry AC Hibbert L Cacalano NA Johnston JA. SOCS2 can enhance interleukin-2 (IL-2) and IL-3 signaling by accelerating SOCS3 degradation. Mol Cell Biol. (2005) 25:9115–26. 10.1128/MCB.25.20.9115-9126.2005 16199887 PMC1265772

[18] Machado FS Aliberti J. Impact of lipoxin-mediated regulation on immune response to infectious disease. Immunol Res. (2006) 35:209–18. 10.1385/IR:35:3:209 17172647

[19] Esper L Roman-Campos D Lara A Brant F Castro LL Barroso A. Role of SOCS2 in modulating heart damage and function in a murine model of acute Chagas disease. Am J Pathol. (2012) 181:130–40. 10.1016/j.ajpath.2012.03.042 22658486 PMC3388166

[20] Brant F Miranda AS Esper L Gualdrón-López M Cisalpino D De Souza DG. Suppressor of cytokine signaling 2 modulates the immune response profile and development of experimental cerebral malaria. Brain Behav Immun. (2016) 54:73–85. 10.1016/j.bbi.2016.01.002 26765997

[21] Machado FS Johndrow JE Esper L Dias A Bafica A Serhan CN Antiinflammatory actions of lipoxin A4 and aspirin-triggered lipoxin are SOCS-2 dependent. Nat Med. (2006) 12:330–4. 10.1038/nm1355 16415877

[22] Tietze F. Enzymic method for quantitative determination of nanogram amounts of total and oxidized glutathione: applications to mammalian blood and other tissues. Anal Biochem. (1969) 27:502–22. 4388022 10.1016/0003-2697(69)90064-5

[23] Marques PE Antunes MM David BA Pereira RV Teixeira MM Menezes GB. Imaging liver biology *in vivo* using conventional confocal microscopy. Nature Protocols (2015) 10:258–68. 10.1038/nprot.2015.006 25569332

[24] Costa VV Fagundes CT Valadao DF Cisalpino D Dias AC Silveira KD. A model of DENV-3 infection that recapitulates severe disease and highlights the importance of IFN-gamma in host resistance to infection. PLoS Negl Trop Dis. (2012) 6:e1663. 10.1371/journal.pntd.0001663 22666512 PMC3362616

[25] Amaral SS Oliveira AG Marques PE Quintao JL Pires DA Resende RR. Altered responsiveness to extracellular ATP enhances acetaminophen hepatotoxicity. Cell Commun Signal. 11:10. 10.1186/1478-811X-11-10 23384127 PMC3608937

[26] Urban TJ Shen Y Stolz A Chalasani N Fontana RJ Rochon J. Limited contribution of common genetic variants to risk for liver injury due to a variety of drugs. Pharmacogenet Genomics (2012) 22:784–95. 10.1097/FPC.0b013e3283589a76 22968431 PMC3636716

[27] Uetrecht J Naisbitt DJ. Idiosyncratic adverse drug reactions: current concepts. Pharmacol Rev. (2013) 65:779–808. 10.1124/pr.113.007450 23476052 PMC3639727

[28] Liang Y Xu W Peng H Pan H Ye D SOCS signaling in autoimmune disease: molecular mechanisms and therapeutic implications. Eur J Immunol. (2014) 44:1265–75. 10.1002/eji.201344369 24595859

[29] Masuzaki R Zhao S Valerius MT Tsugawa D Oya Y Ray KC. SOCS2 balances metabolic and restorative requirements during liver regeneration. J Biol Chem. (2016) 291:3346–58. 10.1074/jbc.M115.703264 26703468 PMC4751379

[30] Zellmer S Sickinger S Schidt-Heck W Guthke R Gebhardt R. Heterogeneous expression of suppressor of cytokine signalling 2 (SOCS-2) in liver tissue. J Anat. (2009) 215:176–83. 10.1111/j.1469-7580.2009.01085.x 19470084 PMC2740965

[31] Reilly TP Bourdi M Brady JN Pise-Masison CA Radonovich MF George. Expression profiling of acetaminophen liver toxicity in mice using microarray technology. Biochem Biophys Res Commun. (2001) 282:321–8. 10.1006/bbrc.2001.4576 11264010

[32] Numata K Kubo M Watanabe H Takagi K Mizuta H Okada S. Overexpression of suppressor of cytokine signaling-3 in T cells exacerbates acetaminophen-induced hepatotoxicity. J Immunol. (2007) 178:3777–85. 10.4049/jimmunol.178.6.3777 17339476

[33] Marques PE Oliveira AG Pereira RV David BA Gomides LF Saraiva AM. Hepatic DNA deposition drives drug-induced liver injury and inflammation in mice. Hepatology (2015) 61:348–6. 10.1002/hep.27216 24824608

[34] Streetz KL Tacke F Leifeld L Wüstefeld T Graw A Klein C. Interleukin 6/gp130-dependent pathways are protective during chronic liver diseases. Hepatology (2003) 38:218–29. 10.1053/jhep.2003.50268 12830005

[35] Tacke F Luedde T Trautwein C. Inflammatory pathways in liver homeostasis and liver injury. Clin Rev Allergy Immunol. (2009) 36:4–12. 10.1007/s12016-008-8091-0 18600481

[36] Zadjali F Santana-Farre R Vesterlund M Carow B Mirecki-Garrido M Hernandez-Hernandez I. SOCS2 deletion protects against hepatic steatosis but worsens insulin resistance in high-fat-diet-fed mice. FASEB J. (2012) 26:3282–91. 10.1096/fj.12-205583 22562833

[37] Perkins ND Gilmore TD. Good cop, bad cop: the different faces of NF-kappaB. Cell Death Differ. (2006) 13:759–72. 10.1038/sj.cdd.4401838 16410803

[38] Schreiber J Jenner RG Murray HL Gerber GK Gifford DK Young RA. Coordinated binding of NF-kappaB family members in the response of human cells to lipopolysaccharide. Proc Natl Acad Sci USA. (2006) 103:5899–904. 10.1073/pnas.0510996103 16595631 PMC1426243

[39] Jamaluddin M Wang S Boldogh I Tian B Brasier AR TNFalpha-induced NF-kappaB/RelA Ser(276) phosphorylation and enhanceosome formation is mediated by an ROS-dependent PKAc pathway. Cellular Signal. (2007) 19:1419–33. 10.1016/j.cellsig.2007.01.020 17317104

[40] Watkins PB Kaplowitz N Slattery JT Colonese CR Colucci SV Stewart PW. Aminotransferase elevations in healthy adults receiving 4 grams of acetaminophen daily: a randomized controlled trial. JAMA (2006) 296:87–93. 10.1001/jama.296.1.87 16820551

